# Estimating view parameters from random projections for Tomography using spherical MDS

**DOI:** 10.1186/1471-2342-10-12

**Published:** 2010-06-18

**Authors:** Yi Fang, Sundar Murugappan, Karthik Ramani

**Affiliations:** 1 School of Mechanical Engineering, Purdue University, West Lafayette, IN, 47907, USA; 2 School of Electrical Computer Engineering (by courtesy), Purdue University, West Lafayette, IN, 47907, USA

## Abstract

**Background:**

During the past decade, the computed tomography has been successfully applied to various fields especially in medicine. The estimation of view angles for projections is necessary in some special applications of tomography, for example, the structuring of viruses using electron microscopy and the compensation of the patient's motion over long scanning period.

**Methods:**

This work introduces a novel approach, based on the spherical *multidimensional scaling (sMDS)*, which transforms the problem of the angle estimation to a sphere constrained embedding problem. The proposed approach views each projection as a high dimensional vector with dimensionality equal to the number of sampling points on the projection. By using SMDS, then each projection vector is embedded onto a 1D sphere which parameterizes the projection with respect to view angles in a globally consistent manner. The parameterized projections are used for the final reconstruction of the image through the inverse radon transform. The entire reconstruction process is non-iterative and computationally efficient.

**Results:**

The effectiveness of the sMDS is verified with various experiments, including the evaluation of the reconstruction quality from different number of projections and resistance to different noise levels. The experimental results demonstrate the efficiency of the proposed method.

**Conclusion:**

Our study provides an effective technique for the solution of 2D tomography with unknown acquisition view angles. The proposed method will be extended to three dimensional reconstructions in our future work. All materials, including source code and demos, are available on https://engineering.purdue.edu/PRECISE/SMDS.

## Background

This work studies the problem of 2D tomography with unknown view angles and discusses the potential applications of our work. We give the background of our work, reviews of the existing methods and a brief introduction of our proposed method in the following subsections.

### Tomography with unknown view angles

The computed tomography has been successfully applied to various fields over the past decades, for example, medical imaging, synthetic aperture radar (SAR) and Cryo-electron microscopy (cryoEM) for structuring viruses [1-4]. The traditional tomography is defined as a process of recovering the object from the measurements that are line integrals of that object at some set of known orientations (view angles). However, in some special situations, obtaining the view angles is difficult or suffers from accurate measurement. For example, 1) the patient's motion owing to long scanning period can result in uncertainty of view angles, 2) the data acquisition of single particle cryoEM are the line integrals of many identical copies of virus molecules at random orientations. Therefore, the research of a more generalized tomography independent of known acquisition view angles is worthwhile to study for the reconstruction of the objects under various circumstances. Our overarching goal is the reconstruction of 3D virus from 2D cryoEM images. As one step towards the goal, we address the problem of image alignment (orientation determination). In the application of cryoEM, the projections for the macromolecules at a preset angle are captured for a large number of identical macromolecules at different unknown and random orientations. If we assume there is no overlapping o the projection, the imaging process is the same as having projections of the macromolecule at multiple but unknown angles while fixing the position of a single homogeneous macromolecule [5]. Hence, like [5-7], we propose a computational model which is equivalent to the real-world scenario, as having projections at different, unknown and random angles while fixing the position of a single homogeneous macromolecule.

Recently, the uniqueness and feasibility of tomography with unknown view angles has been discussed in [3,8]. An object can be uniquely determined using the projection data with a certain number of unknown and distinct view angles. The reconstructed object is subjected to a global arbitrary spatial rotation, which doesn't influence the study of the investigated objects. A review of recently proposed methods dealing with this issue is described below.

### Review of existing methods

Several approaches for analyzing projections measured from unknown view angles have been developed over last two decades [6,8-18]. Those methods can be roughly categorized into two classes, iterative and non-iterative methods. The iterative methods described in [8,18] use the moment characterization of the range space of the Radon Transform, known as the Helgasson-Ludwig (HL) consistency conditions to reconstruct the image. The authors proposed a Bayesian approach for the view angle estimation for tomography in [16]. An integrated statistical technique for volume reconstruction with unordered sequential slices is presented in [17]. The limitations of the iterative methods cited above arise because of a huge computation complexity owing to the solution of a large nonlinear problem at each iteration [5]. Recently, non-iterative approaches have been developed in [5,6,13] to achieve a fast way of structuring the object from its projections with unknown view angles. Yagle introduced a simple non-iterative algorithm based on circular harmonic expansion in [5]. The work decouples the view angles estimation problem from the image estimation problem and thereby largely reduces the computational expenses. Two manifold learning based techniques showed great performance at solving the view angles uncertainty [6,13]. Georg etc. [13] applied the manifold learning to sort the time-ordered slab data for automatic estimation of lung volume without any external breath measurements. The goal is to piece together the local slab data on a proper position in a globally consistent manner, with regards to breath phase circle. Coifman et al. [6] presented a Laplacian graph based manifold learning method to enforce the view angles embedded on the circle. All of the non-iterative approaches have a common attractive property: computation is really efficient as the angle estimation problem is transformed into a matrix eigenvalue problem with the size equal to the number of angles.

### sMDS for view angles uncertainty

The manifold learning methods show attractive properties in terms of estimating view angles from the acquisition data [6,13,19]. As mentioned in [19], the popular manifold learning methods such as, Multidimensional scaling (MDS) [20], ISOMAP [21], and locally linear embedding (LLE) [22], cannot handle the view angle uncertainty problem directly because these methods can only embed points in a flat space while the view angles are intrinsically distributed on a sphere. To solve this problem, our study introduces a reconstruction scheme based on spherical MDS (sMDS) proposed in [23], which is able to embed points on a spherical manifold. Our work is inspired by the projection-slice theorem, which states that the Fourier transform of the projection of a 2D function is equal to a slice through the origin of the 2D Fourier transform of that function. The algorithm for estimation of view angle for each projection consists of three steps. First, Fourier transform is applied to all of the projection data (e.g.  denotes the Fourier transform of the *ith *projection). Then the distance between the pairwise Fourier transform (e.g.  and ) is measured to build the distance matrix. Last, sMDS is applied on this distance matrix to estimate the view angle for each projection data. The algorithm details are presented in Section 2. Essentially the embedding process assigns each projection a point in a low dimensional intrinsic parameter space. This process assembles the similar projections that are close to each other without using any prior knowledge. There are two reasons to build the distance matrix in the Fourier domain 1) it is more flexible to perform the computations on the Fourier domain as it helps us to handle noise by allowing us to choose a proper range of frequency instead of the complete range. This is even useful when the noise level is high as the signal in low frequency range is comparable to noise while the signal in high frequency range is totally buried by noise. Therefore, the distance computed in the Fourier domain is more robust to noise by controlling the range of frequency in Fourier domain. The algorithm for choosing an optimal range in Fourier domain is one of our future work. Currently, we use either half of the range or the full range itself if noise is low. 2) the magnitude of the Fourier vector is invariant to the center shift of the image. Thus, even when the image shifts during projecting, the distance would not be affected if the computation is only based on the magnitude of the Fourier values.

**Figure 1:**
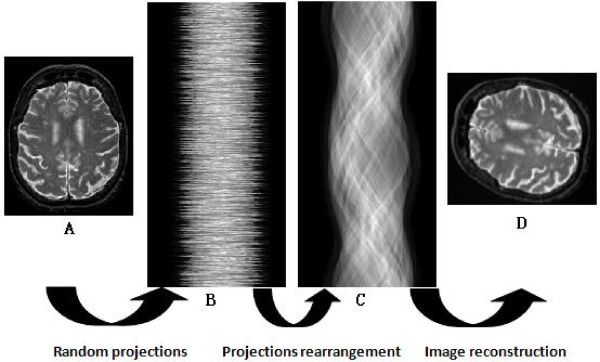
**Reconstruction flowchart**. The flowchart shows three basic steps of the reconstruction procedure from projections data with unknown view angles. Figure (A) is the original brain MR image. Figure (B) shows the projections data generated by projecting the brain image from a set of view angles randomly. All of projection data stack up along the vertical direction. We can see from the figure that the order of the projection data is really shuffled due to the random projection angels. Figure (C) shows the sorted projection data, which is also named sinogram. The comparison between Figure (B) and Figure(C) demonstrates the capability of our method for sorting the projection data. Figure (D) display the reconstructed image from the sorted projection data by using the inverse radon transform. The reconstructed image is subject to a global rotation transform of the original image.

The contributions of the proposed method are the development of a sMDS based scheme for embedding the projection data onto a 1D sphere and then orienting the projection data using the coordinates of the embedded points. The major portion of our contributions is the rearrangement of random projections data, which is illustrated in the steps from Figure 1(B) to 1(C). Figure 1 shows the basic framework and proposed idea. The projections are first generated from a set of view angles randomly, the projection data are then sorted in a globally consistent manner, and the reconstruction of the image from the ordered projections is completed through the inverse radon transform.

## Methods

### Spherical MDS

Given the pairwise distances between points, multi-dimensional scaling is widely adopted to embed these points in a low dimensional space which are consistent with pairwise distances. The main goal of the embedding techniques is to unveil the structure underlying a set of objects under investigation, for instance, images. However, the widely used embedding approaches such as MDS, LLE, and ISOMAP [21,22], only solve the embedding of the high-dimensional points in flat space like a plane. These techniques would fail in the case where the intrinsic structure of the manifold is topologically not a plane. The sMDS presented in [23] expands the applicability of MDS for embedding the points on a sphere. There are two aspects which makes sMDS different from MDS. First, the sMDS measures the pairwise distance between points using geodesic distance instead of the Euclidean distance because the points lie on a sphere. Second, the method of constructing the centering matrix, which transforms the distance matrix into dot-product form, is different from the MDS owing to the distance measurements in the non-Euclidean space. The algorithm procedure for sMDS for embedding points onto the k-dimensional sphere described in [23], is as follows.

1. Build the pairwise distance matrix **M**, and

2. Compute the dot-product form of the distance matrix , where Γ denotes a operator applied on the distance matrix **M **and *r *denotes the radius of the sphere calculated using , and

3. Choose the first the *k *+ 1 eigenvectors of the distance matrix Γ(**M**). The *ith *eigenvector is denoted as  = (*V*_*i*_(1), *V*_*i*_(2)), ..., *V*_*i*_(*n*) where n is the number of the points. The coordinate of the *jth *embedded point is  = (*V*_1_(*j*), *V*_2_(*j*), ..., *V*_*k*+1_(*j*)) where *k *is the dimensionality of the sphere.

This procedure briefly introduces the general ideas about the sMDS. The details about how to apply sMDS for 2D tomography application is provided in the following sections.

### Problem definition

#### Fourier slice theorem

**Figure 2:**
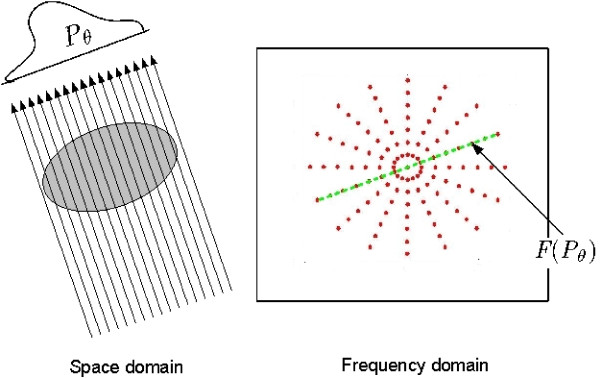
**Illustration of Fourier slice theorem**. The Figure illustrates the basics of Fourier slice theorem. The left figure shows the simulation of generating the projection from the view angle *θ*. The **P**_*θ *_denotes the projection from view angle *θ*. The right figure shows the Fourier transform of the image in the left. The green marked slice and the Fourier transform of **P**_*θ *_are equal according to the Fourier slice theorem.

The Fourier slice theorem is the fundamental theory behind tomography. The theorem states that the one-dimensional Fourier transform of a parallel projection is equal to a slice through the origin of the two-dimensional Fourier transform. It opens up the probability to reconstruct the object via performing the inverse Fourier transform. Figure 2 is a graphical illustration of the projection slice theorem in two dimensions. The left figure shows one projection (*P*_*θ*_), from a view angle, mathematically, an integral of the object density function along the sight parallel line. The figure on the right shows the two dimensional fourier transform of the object. The green color points represent a slice of 2D Fourier transform and the projection *P*_*θ *_is a one-dimensional Fourier transform pair according to the theorem.

#### Derivation of the Model

##### Projection and its Fourier transform

Each projection data is viewed as a high dimensional vector (PVector)  = (*P*(*t*_1_), *P*(*t*_2_), ..., *P*(*t*_*n*_)) where *t*_1_, *t*_2_, ..., *t*_*n *_are equally spaced sampling points on the projection data and *n *is the dimensionality of the vector. The Fourier transform of the PVector is also viewed as a high dimensional vector (FTVector), represented as (*F*(*ρ*_1_), *F*(*ρ*_2_), ..., *F*(*ρ*_*n*_)) where *ρ*_1_, *ρ*_2_, ..., *ρ*_*n *_are represented as equally spaced sampling points on the Fourier transform of the projection data and *n *is the dimensionality of the vector. According to the theorem, each projection collected from unknown view angle has a unique Fourier transform pair, which is a slice of the 2D Fourier transform of the object. Therefore, the problem of sorting projections in space domain is equivalent to orienting their corresponding Fourier transforms in frequency domain.

##### Orienting Fourier transforms

The rearrangement of Fourier transforms is essentially a dimensionality reduction problem with the internal structure constrained by spherical manifold. As we can see in Figure 2, the slices are distributed along the radial direction. We can reason analytically and imagine that a FTVector (a slice),  is intrinsically restricted on a circle since a line passing through the origin could be uniquely parameterized by its orientation. We provide an observation in the appendix section to explain why the underlying structure is a 1D sphere (a circle). Mathematically, the FTVector  = (*F*_*i*_(*ρ*_1_), *F*_*i*_(*ρ*_2_), ..., *F*_*i*_(*ρ*_*n*_)) could be intrinsically reduced to a two dimensional point  = (*X*_*i*_, *Y*_*i*_) on a circle, where i denotes the *ith *projection and *X *and *Y *denote the principal axes in cartesian coordinate. The FTVectors can be oriented based on the corresponding two dimensional point set {, *i *∈ (1, 2, ..., *n*)} where n is the number of points.

##### Orienting the projections

As we discussed above, one projection is uniquely associated with a slice in the 2D Fourier transform space. The low dimensional intrinsic parameter for each FTVector could be assigned to the corresponding PVector directly. The embedded point set reveals the orientation of the projection in a coherent global manner, which clearly organize the projections with unknown view angles. Note that the estimated organization is subjected to a global arbitrary rotation, which doesn't influence the analysis of the reconstruction result.

This model basically converts the view angle uncertainty problem to a dimensionality reduction problem and the reduced low dimensional structure is used to recover the view angles.

### View angles estimation algorithm

#### Pairwise distance matrix

In this work, the pairwise distance matrix is built by following the steps described in [21]. The computation of the pairwise distance matrix consists of two steps. First, given a distance threshold **T**, the high dimensional points (e.g. the FTVector in this work) which are neighbors, are determined based on distance *d*(*A*, *B*) between pairs of points *A*, *B *( Eq.1). A particular point connects to other points if the pairwise distance d(A, B) is less than the predefined threshold **T**. A weighted graph **G **with weight *d*(*A*, *B*) between neighboring points can be used to describe these neighborhood relations. Second, the Dijkstra's algorithm [24] is applied to the weighted graph **G **to compute the shortest path distance in the graph, which well approximates the geodesic distance between all pairs of the points.

Note that there have been a number of standard ways of measuring the distance between two high dimensional vectors as described in [25]. The popular standards include *L*1 norm, *L*2 norm, λ^2 ^measure, and Bhattacharyya distance. In this work, we adopt a widely used distance measurement, *L*2 norm.(1)

where  and  denote differently oriented FTVector respectively.

#### Estimation of view angles

##### Determination of coordinates

The eigenvalue and eigenvector of the dot-form matrix, Γ(**M**), intrinsically reflect the relative positions of the embedded points in a globally consistent way. The coordinates of the embedded points are solved by the following procedure.

1. Rank the eigenvalues of the Γ(**M**) in decreasing order and choose first two eigenvectors,  and , corresponding to the first two eigenvalues, and

2. Normalize each pair (*V*_1_(*i*), *V*_2_(*i*)) to a unit vector, where *i *is the index of the embedded points. The normalized vectors will be used as the coordinates of the embedded point  on the circle.

##### Determination of view angles

For determination of view angles, we first compute an initial estimate of view angles by the inverse triagonometric functions, arctangent. We then use a refinement process to obtain the final accurate angles.

1. Apply the inverse triagonometric function, arctangent, to the coordinates of embedded points for calculating the initial set of view angles,(2)

where i denotes the *ith *point, *Y *_*i *_and *X *_*i *_denote the coordinate of the *ith *embedded point and *φ*_*i *_the estimated view angle for the *ith *projection.

2. Sort the initial set of view angles *φ*_1_, *φ*_2_, ..., *φ*_*n*_) in ascending order, uniformly rearrange the view angles along the circle, and associate the refined view angle set to the original projection data.

## Results

We have implemented sMDS for 2D tomography with unknown view angles and assessed its performance from the experimental results. The algorithms presented in the paper are implemented on a Pentium D 3.2 GHz computer with 1 G RAM running Windows XP. The images for the experiments have been chosen from the Whole Brain Atlas http://www.med.harvard.edu/AANLIB/home.html. The Whole Brain Atlas provides a large set of MR images for both normal and diseased brain. There are three parameters in the experimental setting, the number of the projections, **N**, the threshold, **T**, which is used to determine the neighborhood relation between a pair of points, and the number of the sampling points along each projection, **S**.

### Orienting projection data

**Figure 3:**
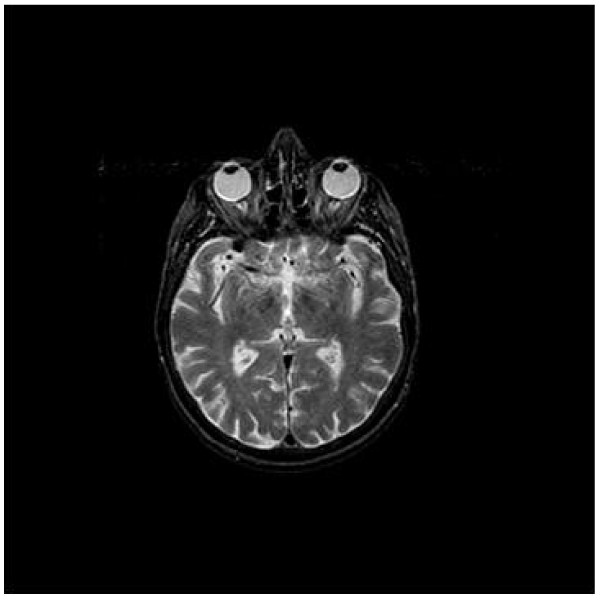
**Brain MR image**. Figure displays a normal brain MR image. The image is downloaded from the Whole Brain Atlas, Harvard University.

**Figure 4:**
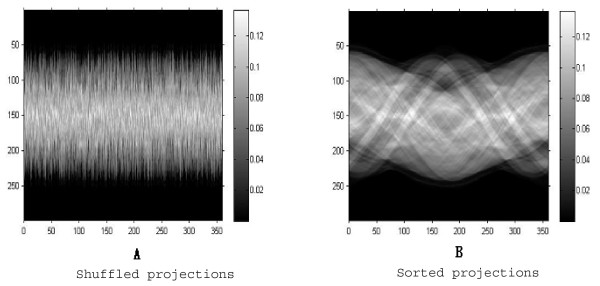
**Random projections and its rearrangement**. Two figures display the projections generated from a set of view angles. The horizontal axis denotes the index of projections and the vertical axis denotes the sample positions of each projection. As we can find, the range of horizontal axis from 0 to 360 indicates that there are total 360 projections in this figure, and the range of vertical axis from 0 to 300 indicates that there are 300 sampled points on each projection. The colorbar on the right side indicate the value of projection data. The projection data in the Figure (A) and Figure (B) are the same set, but the difference between two figures is that projections in (A) are unordered and sorted in (B). The figure (B) is a common named sinogram, produced by the radon transform of the image. Comparison between two figures demonstrates the performance of our method in rearranging the randomly produced projections.

In the first experiment, we verify the performance of the sMDS. This test starts with generating random projections from a set of view angles. The number of the projections, **N**, is set at 360 and the number of the sampling points of each projection is set at 299. We used a normal brain MR image as the experimental object, shown in Figure 3. Figure 4 shows the visualization of the projection data. The Figure 4(A) illustrates the projections with random view angles and Figure 4(B) shows the sorted projection data. The horizontal axis in the figure is labelled with the index of projection data, and the vertical axis of the histogram is the number of the sampling points of each projection. The colorbar on the right side indicates the value of the projection data at each sampled position. The value range of horizontal axis is between 1 and 360, indicating that there are total 360 projections acquired from different view angles. The value range of vertical axis is from 1 to 299, indicating that number of the sampling points, **S**, set at 299 for each projection data.

A visual comparison between the two figures demonstrates that sMDS sorts the projection data well. We can see from the Figure 4(A) that the projection data are shuffled owing to the random projections, while in the Figure 4(B) the projection data are clearly ordered with the global relative orientation.

### Verification of reconstruction quality

**Figure 5:**
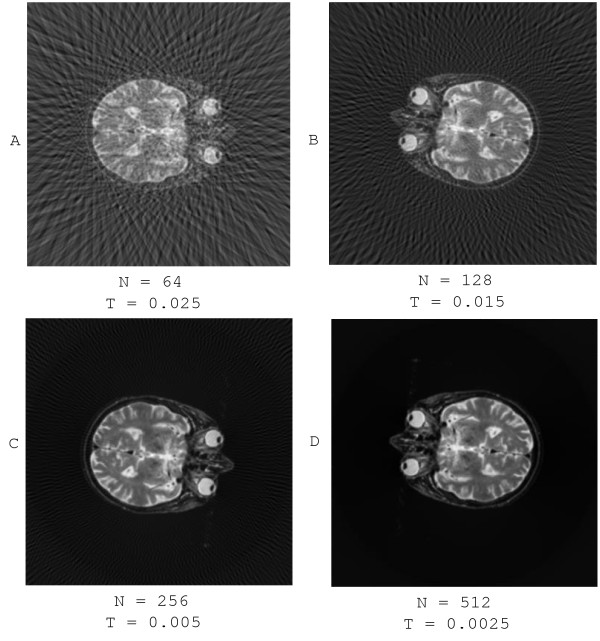
**Reconstruction results**. The reconstructed images from the projections, which are generated by projecting Figure 3 through different view angles. N and T in the figure denote the number of projections and threshold for neighbor relation determination respectively. As we can see, the reconstruction quality is enhanced with the increase of the number of the projections. Note that the threshold would be adjusted with the change of number of projection as the more the projection the less the distance between pairwise projections.

In the second experiment, we verify the performance of sMDS for view angles estimation. We generate different number of the projections of a normal brain MR image from a set of view angles ranging from 0°to 360°. We demonstrate the performance of our proposed method from the comparison between the original and reconstructed images. We test the effect of the projection number, **N**, and threshold value, **T**. Figure 5 shows reconstructed results of the brain MR image (see Figure 3) using different number of projections. The threshold, **T**, used for the neighbors detection are slightly varied according to the number of the projections. With the increase in the number, **N**, the distance metric between pairwise points decrease. Therefore, we need to lower the value of the threshold to assure that a proper neighbor relation for each projection is preserved. The reconstruction quality is enhanced with the increase in the number of the projections(see Figure 3). In addition, we observe that the reconstructed images are subjected to an arbitrary rotation of the original image. We further verify quantitatively the reconstruction performance by comparing the original image with the reconstructed image. In our test, we cannot directly subtract the original from the reconstructed image as there is an arbitrary rotation of the reconstructed image. However, the registration of the two images can remove the effect of the arbitrary global rotation. In this experiment, the registration between original and reconstructed images is trivial as the correspondence between the randomly shuffled projections and the re-organized projections can be easily tracked. The global rotation angle can be easily retrieved by finding the difference between any corresponding pairs. For example, we can track the first projection of the original image, find its relative position in the re-organized projections sequence, then compute the rotation angle. We provide two quantitative measures for the reconstruction performance: one is the peak signal-to-noise ratio (PSNR), and the other is mean squared error (MSE), which are calculated using Eq.3 and Eq.4.(3)(4)

**Figure 6:**
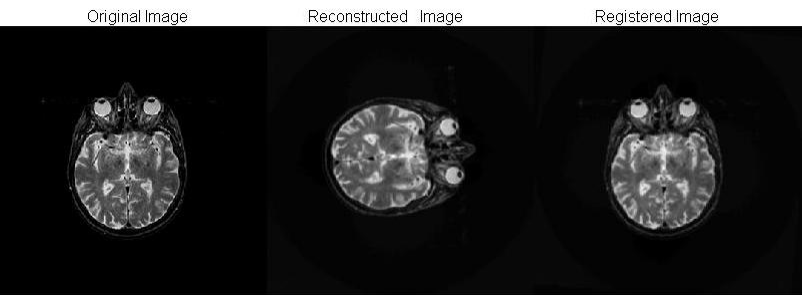
**Original image, reconstructed image and registered image**. The reconstructed images from the projections, which are generated by projecting Figure 3 through different view angles. There are 512 projections generated uniformly and are randomly shuffled. The image on left is the original image, the middle one is the reconstructed from the random projections and the image on the right is the registered image.

Figure 6 shows three images. The image on the left is the original image, the middle image is reconstructed from 512 projections and the image on the right is registered by the method described above. The MSE and PSNR between the original image and registered images are 0.0037 and 24.2804, which show a high quality of reconstruction from the original projections.

**Figure 7:**
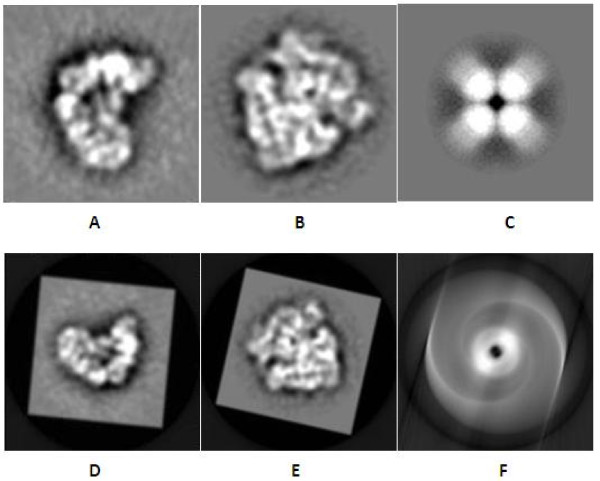
**The reconstruction results of the 2D cryoEM projections**. The figure shows the reconstruction results from the 2D cryoEM projections. Figure (A-C) are the original cryoEM projections. Figure (D-F) are the corresponding reconstruction results of Figure (A-C) respectively.

We further applied sMDS method to the electron microscopic (EM) images. We used a similar procedure as the above for the experiment. The projections are available on the EM database EMDB http://www.pdbj.org/emnavi/. We tested our method on three different density map (EMDB ID:1592,1665 and 5141). Figure 7(A-C) show the original projections from three different objects and Figure 7(D-F) are the corresponding reconstructed images respectively. From these images, we can find that the image can be reconstructed with a high quality if the images are not symmetric. Figure 7(F) shows an unsuccessful reconstruction, due to the inherent symmetry, a limitation of our sMDS method.

### Resistance to noise

**Figure 8:**
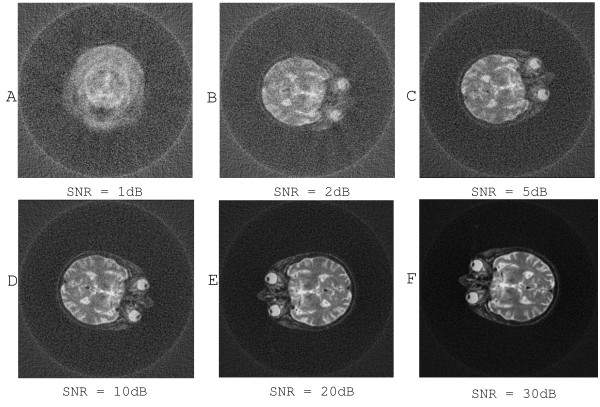
**Reconstruction from noisy projections**. There are six figures displaying the reconstruction results from noisy projections. The projections in different figures are corrupted by the noise to different extent. The SNR underneath each figure indicates the signal noise ratio. We can conclude, from the observation and comparison of the six reconstructed images, that our method is tolerable to noisy data. The reconstruction quality seems reasonable even when the SNR is around 2 dB, in Figure (B).

In the third experiment, we tested the capability of our proposed method in handling projections with additive noise. We choose the brain MR image (see Figure 3) as the experimental subject. We generate a set of projections from 512 view angles randomly, and add noise to the recorded projections. We use Gaussian noise with zero mean and a standard deviation determined by the following equation.(5)

where Signal and Noise are the variance of the noiseless projections and the noise respectively. Figure 8 shows the reconstruction results from the noisy projection data. Our proposed method demonstrates good performance for the noisy projections. From the Figure 8(D-F), we can find that the reconstruction quality is only slightly affected by the noise if the value of the SNR is above 10 dB. However, in the case of SNR less than 2 dB, the noise significantly affects the quality of reconstructions.

## Discussion

### Analysis of experimental results

The experiments conducted in this work verify the performance of our proposed method in dealing with the 2D Tomography with unknown view angles. The number of the projection data **(N) **is one of the most important parameters in our experiments. The manifold learning methods generally require a sufficient number of samples. To meet this requirement, **N **should be relatively big enough, e.g. 256. Secondly, the threshold for neighbors detections is extremely important for our method. Since the sMDS is essentially a method piecing together the local information in a global manner, the local or neighbor relations between points ultimately determine the global embedding. In our experiment, the setting of the threshold is mainly based on **N**. As we understand, larger the value **N**, less the distance between them, and thus a smaller threshold is chosen to build a proper set of neighbors.

### Applications

The estimation of view angles for projections is necessary in some special applications of tomography. We discuss the potential applications of our proposed method in this section. Since the main contribution of this work is the tomography with unknown view angles, this work would be crucial to address the needs of patients in designing next generation tomography equipment. Various studies have shown that it could make the analysis less complicated if the patients are at ease and less anxious. Nowadays, the patients are required to remain motionless during a long scanning period. The difficulty of being motionless and the increasing discomfort and anxiety of the patients lead to the measurement error and analysis complication. Our method shows great potential to minimize these constraints. In addition, there are some situations where the view angles or the acquisition positions of projections are really hard or not possible to be known to us, for instance, the macromolecule structure determination by electron microcopy. Our proposed method offers a very promising approach to reconstruct the object efficiently.

### Limitations

The estimation of view angles or acquisition positions could be categorized as the inverse problem, which is defined as the inference of model parameters from the observed data. There is an unavoidable limitation of methods for the estimation view angles, including the method presented in this work. A perfectly symmetrical object would lead to the fact that a certain number of projections from different view angles are identical to each other. In this case, it is not possible to associate the projections with different view angles because the projection data themselves are not distinguishable, even though they are obtained from different view angles. To overcome this problem, the pre-estimation of the symmetry of the investigated object could be combined with the view angles estimation procedure. There exist some works for detection of the symmetry of an object in computer vision, image processing and computer graphics areas [26-28]. Our future work is to extend our method to three dimensional tomography with unknown view angles and combine the symmetry detection for the symmetrical object reconstruction.

## Conclusions

The uniqueness and feasibility of tomography with unknown view angles have been proved in the earlier works, which offer the theoretical fundamentals for our method. We have introduced an efficient reconstruction procedure for 2D tomography with unknown view angles by means of sMDS. The experimental results indicate our method performs well with high quality image reconstruction, even in the case of highly corrupted noisy data. Our method would potentially provide an alternative approach in dealing with special cases of tomography with unknown acquisition parameters.

## Observation

**Figure 9:**
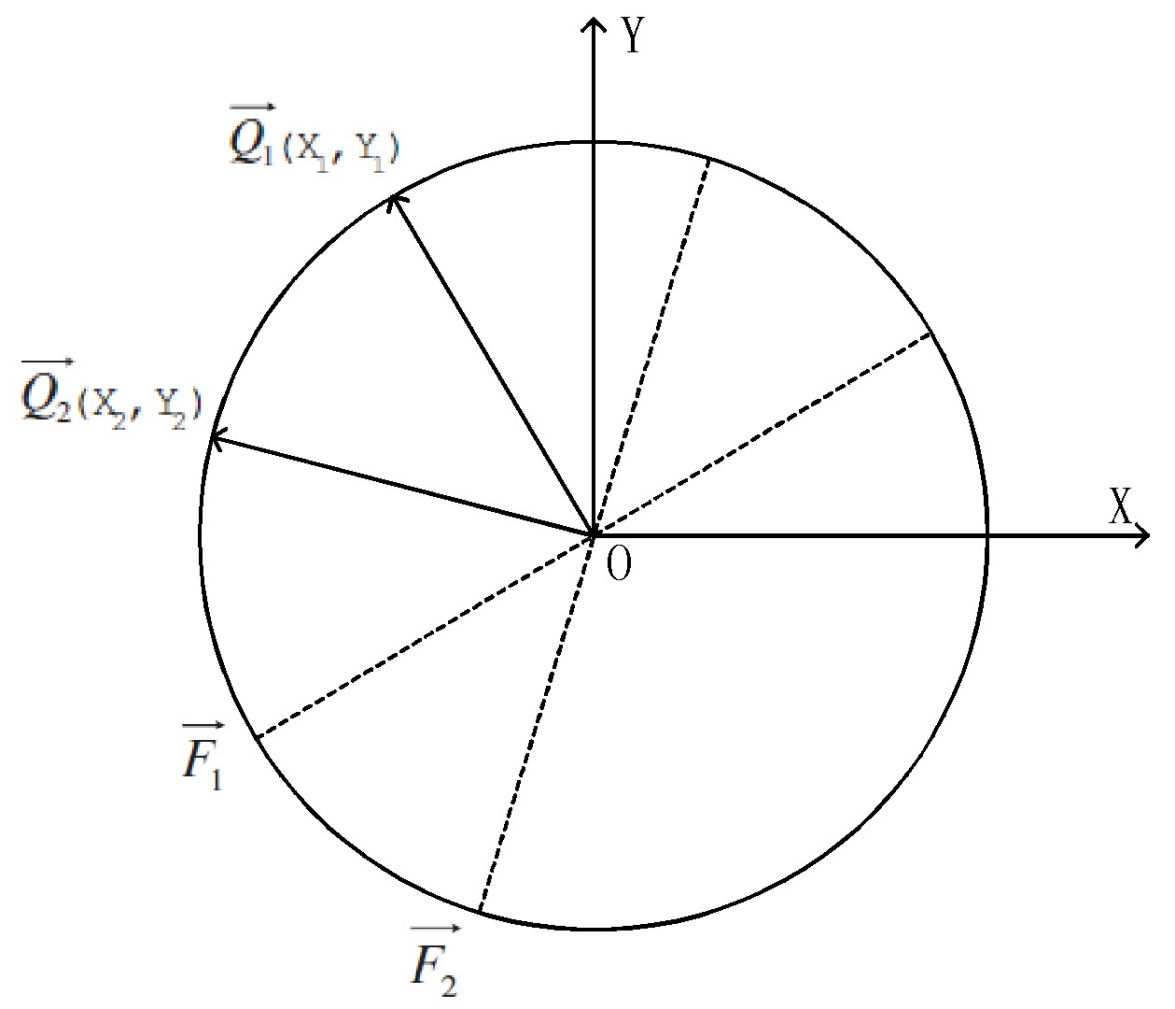
**The illustration of dimension reduction**.

We made the following analytical statement based on the observation from Figure 9. The  and  are two FTVectors with dimensionality equal to the number of the sampled points (the dots). One of the major goals of the manifold embedding methods is to embed the high dimensional vectors onto a low dimensional manifold. The principle of existing dimension reduction methods is that the distance between points in the low dimensional space is consistent with those distances in the original high dimensional space. During the transformation, the pairwise distance between points would be preserved optimally. In the figure, the high dimensional points are  and . And the two dimensional points are the  and  which lie on the circle. The lines from the origin to  and  are perpendicular to  and  respectively. The pairwise distance between point  and  is consistent with the distance between the high dimensional vectors  and . In other words, the internal structure for the FTVectors is constrained to 1D sphere, that is a circle. We can map the set of the FTVectors onto the a set of points on the circle. The sorting of the points on the circle can be used to orient the FTVectors.

## Competing interests

The authors declare that they have no competing interests.

## Authors' contributions

YF generated the original idea, carried out the experiments, and wrote the paper. KR discussed the experiments and extensively revised the manuscript. SM participated in the design of the experiments and extensively revised the manuscript All authors have read and approved the final manuscript.

## Pre-publication history

The pre-publication history for this paper can be accessed here:

http://www.biomedcentral.com/1471-2342/10/12/prepub
